# Transactions of the Medical Society of the State of Pennsylvania

**Published:** 1872-03

**Authors:** 


*Transactions of the Medical Society of the State of Pennsylvania, at its Twenty-Second Annual Session,* held at Williamsport, June, 1871.

The transactions of this Society as usual contains much of interest. Many of the papers, present evidence of thought and study and are of interest to the professional reader.

The address of the retiring president, Prof. S. D. Gross, M. D., LL. D., of Philadelphia, is of much interest, and contains many sound and valuable suggestions. We take the liberty of inserting the following extract from the presidents address:

" Whoever has studied the subject must be convinced that quackery is inherent in our very nature; that it constitutes " part and parcel " of our very being. Mankind naturally possess a strong avidity for the marvellous, and a love of novelty often incites them to the commission of acts of which, when sober sense resumes its sway, they are heartily ashamed. We read with horror of the days of witchcraft, and are struck dumb when we find that an English judge, Sir Matthew Hale, hardly two centuries ago, sentenced two women to the gallows for this alleged crime. Is there no superstition, no bigotry in our age? Has not the witchcraft of two centuries ago its counterpart in the spiritualism of our times? If it do not, like its kindred delusion, send people to the scaffold it sends them to the mad-house and to a permature grave. Mesmerism, clairvoyance, and other follies still have their advocates; many persons still firmly believe that disease is a demon that can only be expelled from the system in which it has taken shelter by the power of religion, pow-wowing, or some other mysterious rites; war, with all its horrors and atrocities, is as much as it was in the darkest ages of the world one of the approved resources of so-called civilized nations in the settlement of their disputes; and bigamy, in its most revolting form, is practised under our own eyes, really the only institution on this continent protected by government!

Physicians, like lawyers, divines, and other people, must take the world as they find it. Human nature is much the same everywhere, and it is not an easy matter to cope with its frailties and prejudices, its follies and its crimes, its bigotry and superstition. Cherishing an enlightened trust in Providence, let us calmly and patiently perform our task upon earth, doing all we can to improve the healing art, to dignify and ennoble our profession, and to benefit the human race."

